# Changes in Ultra-Processed Food Consumption and Lifestyle Behaviors Following COVID-19 Shelter-in-Place: A Retrospective Study

**DOI:** 10.3390/foods10112553

**Published:** 2021-10-23

**Authors:** Walter Sobba, Matthew J. Landry, Kristen M. Cunanan, Alessandra Marcone, Christopher D. Gardner

**Affiliations:** 1 Stanford Prevention Research Center, School of Medicine, Stanford University, Stanford, CA 94305, USA; sobbaw4@stanford.edu (W.S.); matthewlandry@stanford.edu (M.J.L.); amarcone@stanford.edu (A.M.); 2 Quantitative Sciences Unit, School of Medicine, Stanford University, Stanford, CA 94305, USA; cunanank@stanford.edu

**Keywords:** ultra-processed foods, shelter-in-place, snacking, COVID-19, diet, lifestyle behaviors

## Abstract

Ultra-processed food (UPF) consumption poses a potential risk to public health and may be related to shelter-in-place orders. This study utilized the level of food processing as a lens by which to examine the relationships between diet, weight change, and lifestyle changes (including cooking, snacking, and sedentary activity) that occurred during regional shelter-in-place orders. This study used a cross-sectional, retrospective survey (*n* = 589) to assess baseline demographics, changes in lifestyle behaviors using a Likert scale, and changes in dietary behaviors using a modified food frequency questionnaire from mid-March to May 2020; data were collected in the California Bay Area from August to October 2020. Foods were categorized by level of processing (minimally processed, processed, and ultra-processed) using the NOVA scale. Stepwise multiple linear regression and univariate linear regression models were used to determine the associations between these factors. Increased snacking was positively associated with a change in the percent of the calories derived from UPF and weight gain (β = 1.0, *p* < 0.001; β = 0.8 kg, *p* < 0.001) and negatively associated with the share of MPF calories consumed (β = −0.9, *p* < 0.001). These relationships have public health implications as interventions designed around decreased snacking may positively impact diet and weight management and thereby mitigate negative health outcomes.

## 1. Introduction

The recent pandemic caused by coronavirus disease 2019 (COVID-19) poses a substantial risk to public health. While stay-at-home orders [1] issued to protect individuals from COVID-19 have limited the spread of the virus [2] and hospitalizations [3], they may impact the lifestyle, dietary behaviors, and physical and mental health of individuals [4,5]. Furthermore, the shift to work-from-home labor practices has led to a decrease in time spent outside the home and may alter daily habits [6,7,8]. Of particular concern during this time are adverse changes to the diet attributable to the challenges posed by the pandemic. One type of adverse change would be an increased consumption of ultra-processed foods (UPF). Ultra-processed foods, as described by the NOVA classification system, “Are not modified foods, but formulations made mostly or entirely from substances derived from foods and additives, with little if any intact [natural] food” [9]. These exist in contrast to minimally processed foods (MPF), which require little modification to become edible, and are distinct from processed foods (PF) that use various types of preservation or cooking methods to enhance taste and durability [9]. Consumption of UPF, whose low cost and convenient qualities have been marketed by corporate food entities [10], leads to increased caloric consumption and weight gain [11]. UPF consumption is a valuable indicator for diet quality given its studied relationship to several chronic diseases. Evidence demonstrates that high UPF intake is associated with an increased risk of weight gain [12,13], type 2 diabetes mellitus (T2DM) [14], cardiovascular disease [15], all types of cancer [16], and all-cause mortality [17,18,19]. It is worth noting that there are several potential benefits of UPF consumption including their reduced cost, potential to provide important nutrients, long shelf-life, and convenience [11].

Presently, multiple studies have aimed to characterize the effect of the COVID-19 pandemic on dietary and lifestyle behaviors, such as cooking, snacking, and sedentary activity. Researchers have investigated the effects of COVID-19 public health measures on diet, lifestyle behaviors [20,21,22,23,24,25,26,27,28], and weight change [20,21,24,25,26,27,28,29]. Studied diet outcomes include specific foods, food groups [20,21,25,26,27,29], and/or scores of diet quality such as the Healthy Eating Index-2015 (HEI-2015) [30] or Mediterranean diet adherence [22,24]. Much of the research in this area has been conducted outside the U.S., with a smaller number of studies analyzing the effects of stay-at-home orders on dietary and lifestyle behaviors in the U.S. general population [20,23,28,31,32]. The current body of research shows a lack of consensus on the effect of stay-at-home orders on diet quality, with some studies reporting an average shift toward increased quality [20,22,30] and others a shift toward decreased [33] quality, while others did not identify an average change despite a substantial proportion of participants reporting either improving or worsening diet quality [25,28,34]. No studies to date conducted within the U.S. have focused on UPF as a primary outcome or indicator for diet quality. Considering the potential detriment to health associated with increased UPF intake, an examination of factors related to UPF is warranted.

The purpose of this study was to utilize the level of food processing as a lens by which to examine the relationships between lifestyle changes that occurred during regional shelter-in-place orders, diet, and weight change. Specifically, we quantified the changes in UPF consumption and examined the lifestyle factors that were associated with these changes. Recognizing the potential detrimental nature of UPF to public health, we aimed to examine the relationships between lifestyle behaviors and UPF consumption to help inform public health strategies and prompt future research into the relationship between lifestyle behaviors and UPF consumption

## 2. Materials and Methods

### 2.1. Study Design and Study Site

This study used a cross-sectional survey design and was administered remotely to participants. Study participants were chosen from a database of 8423 California Bay Area adults that consented to be on an e-mail listserv used for notification of opportunities to participate in research conducted by the Stanford Nutrition Studies Research Group. The inclusion criterium was individuals 18 and over; there were no exclusion criteria. The survey was administered in three phases. The first phase was a preliminary pilot test to assess comprehension of survey items. From the e-mail listserv database, 500 individuals were randomly selected to be invited to complete pilot testing; 88 chose to participate. Based on initial feedback, the wording of specific questions and available responses were modified to improve comprehension. The revised survey was then distributed to 2000 individuals for a second round of pilot testing; 195 chose to participate. Following this phase, new questions were introduced concerning self-reported mental health challenges, the presence of children in the household, and whether or not participants faced adverse life events during the shelter-in-place order. The final revised survey was distributed to the remaining 5969 from the listserv. All participants were briefed with a participant information sheet outlining the procedures and possible risks of the study before consenting to take the survey. No protected health information was collected, and participation was anonymous and voluntary.

### 2.2. Survey Description

This survey utilized a self-administered online questionnaire that was administered via Qualtrics (Qualtrics, Provo, UT, USA; www.qualtrics.com (accessed on 20 August 2021)) and distributed via MailChimp (The Rocket Science Group LLC., Atlanta, GA, USA; www.mailchimp.com (accessed on 20 August 2021)). It consisted of three components (details provided in Section 2.4). Participants were first asked to record anthropometric and demographic information such as height, weight, sex, income level, and education level. Next, participants self-reported any changes in their behavior during shelter-in-place. The third section of the survey was a self-administered, modified, two-part food frequency questionnaire (FFQ).

### 2.3. Data Collection

Data were collected over a two-month period from 26 August until 21 October 2020 using questions asking about participants’ behaviors from mid-March through the end of May, the period of time during which seven California Bay Area counties were ordered to shelter-in-place [35]. This study specifically investigated a shelter-in-place order; although quarantine measures vary dramatically in scope and implementation, they will be hereafter referred to as “shelter-in-place.” Study staff invited participants to take the survey via an introductory email that described the aims of the study and provided a link by which to participate. Potential participants who did not open the email were sent a follow-up reminder 24 h after the initial email. Upon beginning the survey, respondents were allowed one week to complete the survey before entries were saved.

### 2.4. Key Outcome Measures

#### 2.4.1. Demographic, Anthropometric, and Behavioral Measures

Participants were asked to complete a set of 11 questions to gather anthropometric and demographic information on factors such as height, weight, sex, education, race, and household income. To calculate BMI, self-reported weight and height data collected in pounds and inches were converted to BMI in units of kg/m^2^. Weight change was self-reported using 7 discrete answer choices (originally presented in imperial units) to participants as follows: lost > 4.5 kg, lost 2.7–4.5 kg, lost 0.45–2.25 kg, no change, gained 0.45–2.25 kg, gained 2.7–4.5 kg, and gained > 4.5 kg (1 lb = 0.45 kg). Eighteen questions assessed baseline behaviors such as physical activity and smoking status before the COVID-19 pandemic as well as changes in behavior during the shelter-in-place order (Supplementary Methods). Likert scales with five possible options were used for behavioral change questions. For example, participants were asked how frequently they cooked during shelter-in-place and were provided with the following options: “significantly less, moderately less, at equal frequency, moderately more, significantly more”.

#### 2.4.2. Dietary Intake

To estimate reported caloric intake, we followed the methodology of Jay et al. 2019 [36]. The first step in calculating the shift in percent calories across NOVA categories was assessing pre-pandemic diet using an adapted version of the Harvard-Willett FFQ intended to quantify food group consumption (Supplemental Table S1) [37]. For the purpose of minimizing participant burden, individual items from the 178-item Harvard-Willett FFQ were combined to create 21 items that represented a selection of the major food groups of interest. This abridged FFQ was reviewed for content by 12 members of the Gardner Nutrition Studies Lab Group, including registered dietitians and nutrition researchers, in addition to the study team. The modified FFQ was created following the methodology listed in Malan et al. [38]. Participants reported dietary intake using a range of servings per day for each of the 21 items. Possible responses for consumption of each item in servings included “Never”, “1–4 times monthly”, “2–6 times weekly”, “1–2 times daily”, and “3+ times daily”. To calculate calories, the midpoint of the range provided was used, except for the case of “3+ times daily” in which data were operationalized to 4 servings daily (Supplemental Table S2). Next, the caloric density for one serving of each item was calculated (Supplemental Table S3) and multiplied by participants’ consumed servings of each item. Calories for survey items in the same NOVA group (Supplemental Table S1) were summed to determine calorie consumption for each group and percent of total calories for each group.

To assess diet shift during the COVID-19 shelter-in-place order, a new assessment tool was employed. Developed internally by registered dietitians from the Stanford Nutrition Studies Research Group, this questionnaire asked participants to describe their changes in consumption of each item from the modified Harvard-Willett FFQ using a Likert scale. Respondents could respond that their consumption for each item increased or decreased “significantly” or “moderately” during the shelter-in-place order, or that their consumption for that item did not change. Reporting a “significant” increase was coded as a 50% increase, while a “moderate” increase was coded as a 25% increase in the calories consumed for each item. The same process was used for reported decreases. For “no change” responses, calories were maintained at pre-pandemic levels. This system was applied to each FFQ item, thus resulting in data that captured caloric consumption during the shelter-in-place order. Using the same process as the pre-pandemic FFQ, calories were summed by NOVA group and used to calculate percent of total calories for each group. 

Finally, to assess change in percent calories by NOVA group, percent calories pre-pandemic were subtracted from percent calories during shelter-in-place for minimally processed foods (MPF), processed foods (PF), and ultra-processed foods (UPF). These data were used as the outcome measure for diet change. Utilizing an approach that quantified caloric shifts for each FFQ item rather than a simple increase or decrease allowed each item to be properly weighted in terms of its caloric contribution.

### 2.5. Statistical Analysis

All analyses for this study were conducted utilizing the software R (Version 4.0.2, The R Foundation, Vienna, Austria, 2021) in conjunction with RStudio (Version 1.3.959, RStudio Team, Boston, MA, USA 2021). Stepwise multiple linear regression models and univariate linear regression models were conducted independently using the same independent and dependent variables to assess the associations between demographic factors, behavioral factors, and the enumerated outcomes of change in MPF, PF, UPF, and weight. Stepwise multiple regression models added and dropped independent variables in succession to find the model with the greatest fit as quantified by the Akaike information criterion (AIC); the model with the lowest AIC was selected and presented. Both models included continuous independent variables whose data were captured using a discrete Likert scale. The independent variables that were analyzed satisfied all conditions for multivariate normality, homoscedasticity, and multicollinearity and were linearly related to the outcome measures. Two-sided tests were used for association; significance was set at *p* < 0.05. Beta coefficients from stepwise multiple regression models are presented in the text.

## 3. Results

### 3.1. Demographics

Of the 5969 potential participants contacted for enrollment, 812 opened the questionnaire, and 589 participants completed all of the survey, resulting in a response rate of 9.9%. The participants were predominantly female (73.9%), Caucasian (73.7%), living in a home without children (83.4%), and at least college educated (81.3%). Mean (sd) for BMI was 27.33 (5.85). Demographic characteristics are presented in Table 1.

### 3.2. Changes in Lifestyle Behaviors, Diet, and Weight during Shelter-in-Place

A large proportion of the participants changed their behavior during the shelter-in-place order; both healthy and unhealthy behavior shifts were common (Table 2). As expected, >80% of participants reported spending less time outside the home during the shelter-in-place order, confirming that the public health order changed participants’ daily routines. Notably, it was more common for participants to increase snacking, cooking, and sedentary behaviors than to decrease these activities. Reported changes in participants’ lifestyle behaviors and weight are listed in Table 2. On average, participants decreased the share of calories from MPF in their diet and increased the share of calories coming from UPF. Finally, more participants self-reported gaining weight than losing weight; this finding was consistent across weight change categories (0.45–2.25, 2.7–4.5, 4.5+ kg). Changes in the percent of calories contributed by MPF, PF, and UPF are shown in Table 2.

### 3.3. Demographic and Behavioral Factors Related to Diet Change 

**Figure 1:**
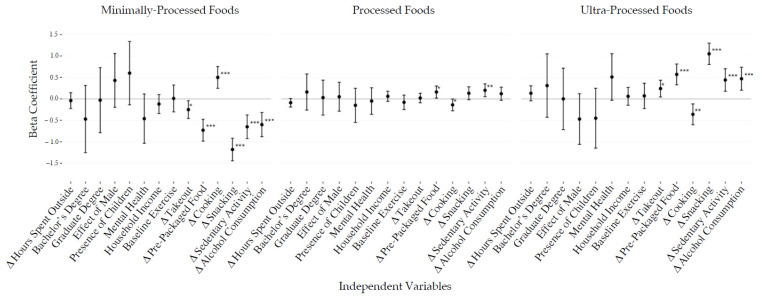
Univariate linear regression outputs presenting the relationships between behavioral, demographic, and anthropometric factors to change in percent calories from minimally processed (MPF), processed (PF), and ultra-processed foods (UPF). Results are presented as beta coefficients with 95% confidence intervals. * *p* < 0.05, ** *p* < 0.01, *** *p* < 0.001.

**Figure 2:**
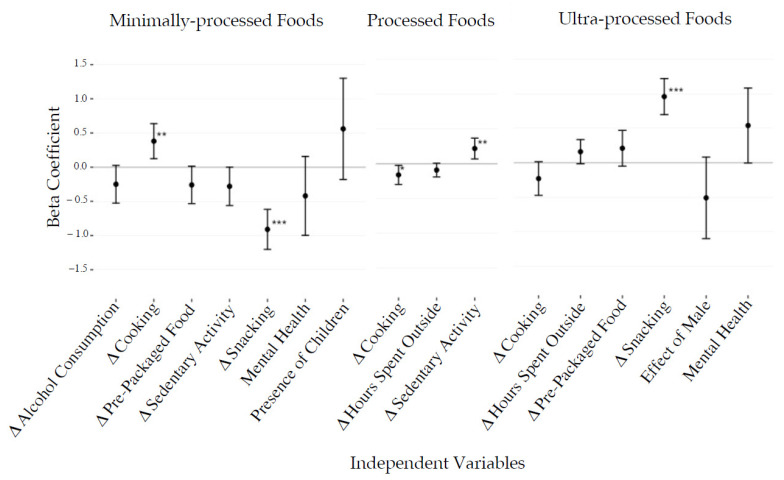
Combined stepwise linear regression outputs presenting the relationships between behavioral, demographic, and anthropometric factors to change in percent calories from minimally processed (MPF), processed (PF), and ultra-processed foods (UPF). This analysis was conducted in six separate models for each type of calorie, separating factors into either demographic/anthropometric factors or behavioral factors. Changes in alcohol consumption, sedentary activity, cooking, takeout food consumption, baseline exercise, and ready-to-eat packaged food consumption were included in the behavioral model, while the remaining variables were included in the demographic/anthropometric model. Factors dropped from models are not shown. Results are presented as beta coefficients with 95% confidence intervals. * *p* < 0.05, ** *p* < 0.01, *** *p* < 0.001.

Changes in calories consumed by level of processing were associated with demographic and behavioral factors (Figure 1 and Figure 2). An increase in snacking was associated with an increase in the share of calories from UPF (β = 1.0, *p* < 0.001) and a decrease in the share of calories from MPF (β = 0.9, *p* < 0.001). Increasing cooking activity was associated with a decrease and an increase in the share of PF and MPF calories, respectively (β = −0.2, *p* = 0.027, β = 0.4, *p* < 0.01). Finally, increased sedentary activity was associated with an elevated proportion of PF calories (β = 0.2, *p* < 0.01). For all univariate and stepwise analysis outputs including beta coefficient values, see Supplemental Tables S4 and S5. 

### 3.4. Anthropometric, Demographic, and Behavioral Factors Related to Weight Change

**Figure 3:**
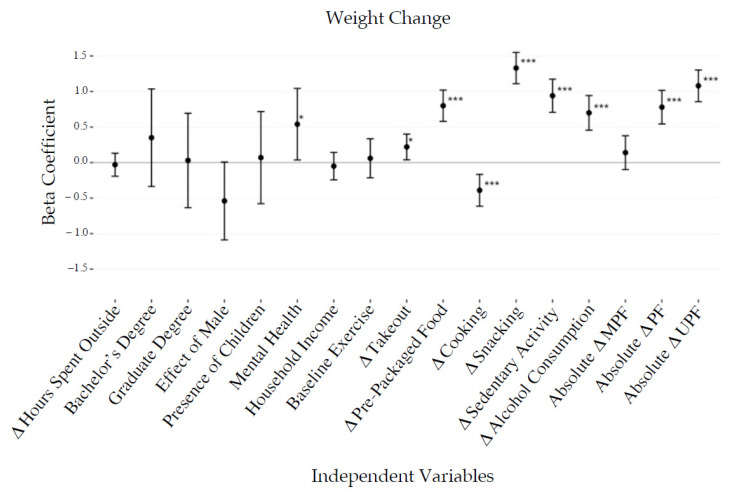
Univariate linear regression outputs presenting the relationships between behavioral, demographic, and anthropometric factors to change in weight in kilograms. Results are presented as beta coefficients measured in kg with 95% confidence intervals. * *p* < 0.05, *** *p* < 0.001.

**Figure 4:**
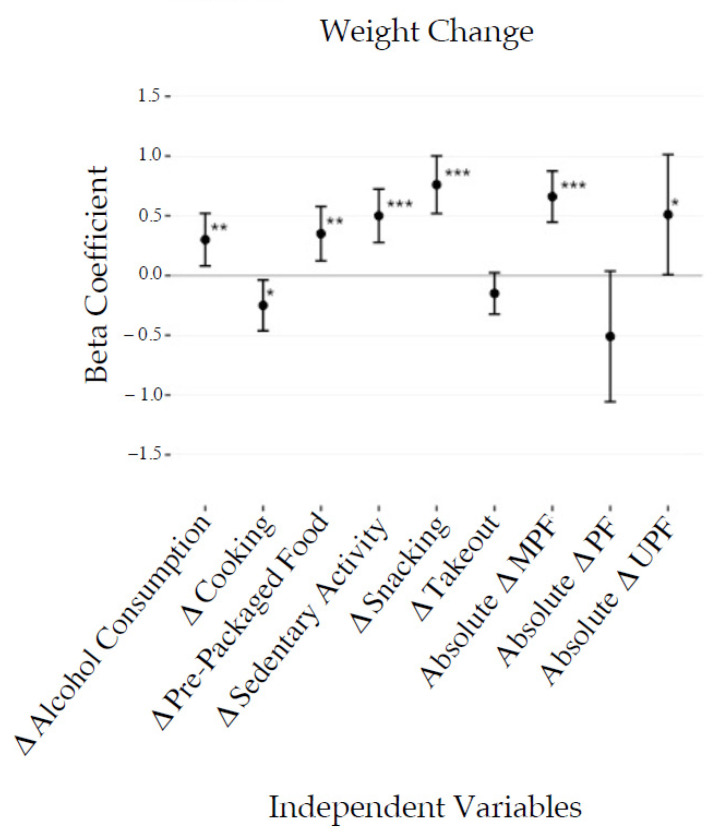
Combined stepwise linear regression outputs presenting the relationships between behavioral, demographic, and anthropometric factors to change in weight in kilograms. This analysis was conducted in six separate models for each type of calorie, separating factors into either demographic/anthropometric factors or behavioral factors. Changes in alcohol consumption, sedentary activity, cooking, takeout food consumption, baseline exercise, and ready-to-eat packaged food consumption were included in the behavioral model, while the remaining variables were included in the demographic/anthropometric model. Factors dropped from models are not shown. Results are presented as beta coefficients in kg with 95% confidence intervals. * *p* < 0.05, ** *p* < 0.01, *** *p* < 0.001.

Weight change, reported by 74.9% of the participants, was associated with anthropometric, demographic, and behavioral variables (Figure 3 and Figure 4). Stepwise multiple regression models revealed that weight gain was associated with a moderate increase in snacking (0.8 kg, *p* < 0.001), sedentary activity (0.5 kg, *p* < 0.001), alcohol consumption (0.3 kg, *p* < 0.001), and ready-to-eat packaged food consumption (0.4 kg, *p* < 0.01), in addition to self-reported mental health challenges (0.5 kg, *p* < 0.05). For every unit of standard deviation increase in the number of UPF calories consumed daily, there was an associated weight gain of 0.7 kg (*p* < 0.001). 

## 4. Discussion

The primary aim of this study was to quantify the changes to California Bay Area residents’ UPF consumption during the COVID-19 shelter-in-place order and determine which demographic and behavioral factors were associated with these changes. In summary, this study found a divergence in health behaviors and outcomes; some participants engaged in more healthy behaviors during the shelter-in-place order, while even more engaged in more unhealthy behaviors. Average UPF calories consumed increased, while MPF calories consumed decreased; more participants gained than lost weight, and a greater share of individuals engaged in unhealthy snacking activity. Increased snacking activity was the strongest factor related to an increase in UPF and a decreased proportion of MPF in the diet, as well as weight gain.

The present body of evidence concerning the effects of shelter-in-place orders on diet shows mixed results. As expected, the differences observed here and reported elsewhere in the literature may reflect the heterogeneous experiences of different individuals, subpopulations, or geographic regions during this pandemic. Public health measures and rates of COVID-19 incidence vary drastically country to country and regionally within a country. A study of Americans during shelter-in-place [20] reported a positive shift in general diet quality. Conversely, a French, study showed a decrease in adherence to the French dietary guidelines mainly attributable to an increase in consumption of processed meat, sweetened beverages, and alcoholic beverages. Other studies conducted in the U.S. [28], the UK [28,34], and France [25] show a divergence in outcomes similar to what was observed in our study, with some participants displaying healthier diet shifts and others reporting unhealthy shifts in their diet [25,28,34]. While changes in the consumption of UPF during shelter-in-place were reported by Deschasaux-Tanguy et al. and Smaira et al., there is a paucity of available data on this perspective of UPF [25,39]. Our sample demonstrated a shift, on average, toward unhealthy diet changes as measured by UPF consumption, though great variation was observed as 46.6% of the participants reported increasing and 36.0% of the participants reported decreasing the share of calories accounted for by UPF. This unhealthy diet shift was echoed in a study of New Yorkers [31] that reported an increase in the energy density—a characteristic of UPF—of female participants’ diets. Our study did not detect different associations of sex on UPF consumption. 

Increased snacking activity was the most strongly related factor to elevated UPF intake. One of two studies that specifically considered UPF consumption during the COVID-19 pandemic reported that those who snacked more frequently during the pandemic were more likely to consume a high amount of UPF before COVID-19 [25]. The other study of Brazilian women found that snacking and “replacing meals with snacks” were linked to increased UPF and decreased MPF consumption [39]. Taken together, these data suggest snacking could factor importantly into a dietary shift toward unhealthy foods. This is further supported by a past analysis of snacking in the U.S. that shows UPF as the primary source of calories in many snack foods [40]. The prevalence of increased snacking, observed in more than a third of the studied sample, echoes previous findings identifying this shift in European [21,26,27,34] and international [23] populations during shelter-in-place orders. 

The effects of snacking were not limited to UPF. An increase in snacking activity was associated with a decrease in the share of MPF in the diet and an increase in weight. The 2015 Brazilian Dietary Guidelines suggest that MPF form the basis of a healthy diet, with special emphasis on plant-based MPF that have a low energy density and high content of dietary fiber [41]. Identifying that snacking displayed the strongest link between decreased consumption of healthy MPF and elevated intake of UPF further contextualizes the robust relationship between snacking and weight gain during shelter-in-place [21,26,28]. While increased snacking was associated with weight gain and observed in almost half of the studied sample, a decrease in snacking was associated with weight loss and improved diet quality. While increased snacking behavior can be viewed as concerning, it also poses an opportunity for intervention. Past research has shown single implementation interventions are effective at curbing snacking behavior [42]. Looking forward to future shelter-in-place situations, further investigation and public health guidance against excess snacking may provide an opportunity to improve diet and limit weight gain. 

Weight gain has been widely reported during COVID-19 shelter-in-place orders [21,23,26,27,34]. In addition to the relationship identified above between increased snacking and weight gain, additional factors that were significantly and positively associated with weight gain were sedentary activity and a positive change in UPF consumption. As previously discussed, the relationship between UPF and weight gain is well documented [12,13]. More than two thirds of the participants reported increasing their sedentary behavior during shelter-in-place, a finding replicated in the U.S. [20,43,44], France [25], and within an international sample [23]. Moreover, out of these studies, the two that analyzed the relationship between sedentary activity and weight gain found that greater increases in sedentary activity were associated with weight gain [20,25]. Self-reported mental health challenges, which reportedly increased during shelter-in-place orders in the U.S. [20], were associated with weight gain in this study, a finding echoed in a prior study [26]. 

The strengths of this study include investigating changes in diet quality using the lens of food processing, which has not been a focus of research during the pandemic and is a topic of growing clinical importance. Furthermore, this study collected data on a range of lifestyle behaviors that were strongly related to changes in diet quality and weight gain.

There were several limitations to this research. First, the participants were drawn from a convenience sample of individuals who agreed to be contacted for nutrition research purposes, which biased the sample toward individuals more interested in nutrition. Additionally, sampling exclusively California Bay Area residents, who were predominantly women and white, limited the generalizability of this study. Second, the survey tool utilized for this study was non-exhaustive and abridged. Thus, the survey did not capture all possible changes in participants’ diets and has not been as extensively validated as the original Harvard-Willett FFQ. To limit participant burden, the modified FFQ was administered once to assess caloric intake of specified food groups before shelter-in-place with options for participants to note which items changed. Thus, the collected data allowed the team to assess trends in diet shift during shelter-in-place though not a specific value to represent caloric change for each participant’s response. However, the survey included food groupings that were carefully selected to represent major contributors to and differentiators of MPF, PF, and UPF, the outcomes of focus in this study. Finally, the measured response rate for our survey was low, <10% [45].

In conclusion, this study identified significant relationships between UPF consumption and recommends that individuals curb snacking activity and sedentary behaviors and increase cooking activity during shelter-in-place. Individuals facing mental health challenges may be especially vulnerable to detrimental health changes that occur during shelter-in-place. Looking forward to potential future shelter-in-place orders, there exists the potential that individuals will further change lifestyle behaviors and spend more time at home [7]. Considering this, future investigations focused on the efficacy and dietary effects of increasing cooking and decreasing snacking behaviors could inform future approaches aimed at mitigating the public health threats that accompany shelter-in-place orders. Furthermore, interventions to support individuals suffering from mental health challenges during shelter-in-place would inform factors affecting diet quality and aid clinicians in improving both diet and mental health outcomes in stay-at-home.

## Figures and Tables

**Table 1:** Participant characteristics.

Question	Response	% (*n* = 589)
Sex	Male	26.1% (154)
	Female	73.9% (435)
Race or ethnicity	Black	2.0% (12)
	Native American	0.3% (2)
	Asian	10.7% (63)
	Native Hawaiian or Pacific Islander	0.2% (1)
	Hispanic	4.8% (28)
	Caucasian	73.7% (434)
	Bi- or Multi-racial	5.1% (30)
	Other	3.2% (19)
BMI (kg/m^2^)	Mean (SD)	27.36 (5.91)
Smoking status	Smokers	2.5% (15)
Parents of children requiring attention	Yes	16.6% (98)
Self-reported mental health challenges	Yes	35.3% (208)
Highest educational attainment	<Bachelor’s degree	18.7% (110)
	Bachelor’s degree	36.5% (215)
	Graduate degree	44.8% (264)
Monthly household income	<USD 1000	2.7% (16)
	USD 1000–2499	5.4% (32)
	USD 2500–4999	16.6% (98)
	USD 5000–9999	30.6% (180)
	USD 10,000–24,999	25.8% (152)
	>USD 25,000	18.8% (111)
Exercise frequency	Never	2.5% (15)
	1–4 sessions per month	11.9% (70)
	1–3 sessions per week	32.1% (189)
	4–7 sessions per week	46.5% (274)
	>7 sessions per week	7.0% (41)

Continuous variables are presented as mean (SD). Categorical variables are presented as % (*n*).

**Table 2:** Changes in lifestyle behaviors, diet, and weight.

Health Behaviors	Changes % (*n*)		
	Increased	Decreased	No Change
Lifestyle Behaviors			
Takeout food	24.1% (142)	52.8% (311)	22.5% (149)
Ready-to-eat packaged food	14.4% (85)	48.9% (288)	47.6% (315)
Cooking	64.3% (379)	10.9% (64)	25.1% (166)
Snacking	36.7% (216)	16.1% (95)	47.3% (313)
Sedentary activity	67.1% (395)	9.7% (57)	22.8% (151)
Hours outside the home	1.5% (9)	82.2% (484)	17.2% (114)
Proportion of Calories Consumed by Level of Processing			
Proportion MPF calories	35.3% (208)	50.4% (297)	14.2% (90)
Proportion PF calories	42.3% (268)	43.3% (255)	14.0% (91)
Proportion UPF calories	49.1% (289)	36.3% (214)	14.2% (92)
Weight			
Weight	48.4% (285)	25.1% (148)	27.4% (181)

## Data Availability

The data that support the findings of this study are available from the corresponding author (CDG) upon reasonable request.

## Supplementary Materials

The following are available online at https://www.mdpi.com/article/10.3390/foods10112553/s1, Supplemental Table S1: Food groups and items included in the original FFQ and adapted FFQ; Table S2: Serving conversion chart from the adapted food frequency questionnaire; Table S3: Caloric density per survey or FFQ item; Table S4: Univariate linear regression outputs; Table S5: Stepwise linear regression outputs. Supplemental Methods: Non-food survey questions.
