# Supplementary material for: Changes in Ultra-Processed Food Consumption and Lifestyle Behaviors Following COVID-19 Shelter-in-Place: A Retrospective Study
Source: Foods. 2021 Oct 23;10(11):2553. doi: 10.3390/foods10112553 (PMC8619493; doi:10.3390/foods10112553)
Supplement: Supplementary file 1 [file foods-10-02553-s001.zip › Supplemental Table S1.pdf]

Table S1. Food groups and items included in the original FFQ and adapted FFQ.

Food group	Original FFQ individual items (178 items)	Adapted, combined FFQ item (21 items)	Item #
Fresh or frozen fruit	Cantaloupe; bananas; avocado; apples or pears; oranges; grapefruit; strawberries (fresh/frozen/canned); blueberries (fresh/frozen/canned); peaches or plums (fresh/canned); apricots (fresh/canned/dried)	Apples; oranges; pears; grapefruit; grapes; berries; melon	1
Minimally processed fruit	Raisins; prunes or dried plums; prune juice; applesauce; apple juice or cider; orange juice (calcium fortified); orange juice (regular); grapefruit juice; other fruit juice; apricots (fresh/canned/dried)	Unsweetened applesauce; unsweetened dried fruits; 100% fruit juices	2
Fruit with added sugar	Strawberries (fresh/frozen/canned); blueberries (fresh/frozen/canned); peaches or plums (fresh/canned); apricots (fresh/canned/dried)	Canned, sweetened berries, citrus, stone fruits; dried, sweetened fruits	3
Vegetables (raw or freshly cooked)	Tomatoes; string beans; broccoli; cabbage; cauliflower; brussels sprouts; carrots (raw); carrots (cooked); corn (frozen/canned); peas or lima beans (fresh/frozen/canned); beans or lentils (baked/dried/soup); winter squash; eggplant, zucchini, or summer squash; yams or sweet potatoes; spinach (raw); spinach (cooked); kale, mustard, or chard; iceberg or head lettuce; romaine or leaf lettuce; celery; peppers; onions (raw); onions (cooked); garlic	Broccoli; carrots; peppers; onions; brussels sprouts; tomatoes; avocado; yams; squash; corn; beans; peas; chard; kale; celery; spinach; romaine; lettuce	4
Vegetables (canned or bottled)	Tomato or V8 juice; tomato sauce; salsa, picante, or taco sauce; corn (fresh/frozen/canned); peas or lima beans (fresh/frozen/canned); mixed vegetables, stir fry, vegetable soup; beans or lentils (baked/dried/soup), ketchup or red chili sauce	Beans; tomatoes; peas; spinach; tomato sauce; pickles; salsa	5
Unprocessed lean protein	Egg beaters or egg whites; omega-3 fortified eggs; regular eggs; chicken or turkey sandwich or frozen dinner; chicken or turkey with skin; liver (chicken or turkey); shrimp, lobster, scallops, or clams; dark meat fish; other fish	Eggs; whole chicken, turkey, fish, seafood	6
Unprocessed red meat	beef, pork, or lamb as a sandwich or mixed dish; pork; beef or lamb; liver (beef, calf, or pork);	Whole beef, pork, game animals	7
Processed meat	Bacon; other chicken or turkey (ground); beef or pork hot dogs; chicken or turkey hot dogs or sausages;	Canned fish; canned seafood; ground beef; ground pork;	8

	salami, bologna, processed meat; other processed meat (sausage); hamburger (lean); hamburger (regular); canned tuna; breaded fish cakes or sticks	bacon; sausage; cured meat; deli meat	
Meat alternatives	Tofu, soy burgers, soybeans, miso, or other soy protein	Tofu, soy chorizo, "beyond meat" product, "impossible burger" product	9
Whole, unprocessed grains and starches	Other grains (bulgur, kasha, buckwheat, etc.); brown rice	Wheat berries, bulgur, quinoa, buckwheat, brown rice, steel cut oats	10
Whole, processed grains and starches	Cooked oatmeal/cooked oat bran; rye or pumpernickel bread; bagels, english muffins, or rolls; whole wheat, oatmeal, or other whole grain bread; white rice, pasta; tortillas; potatoes (baked/boiled/mashed);	Whole wheat bread; whole grain tortilla or bagel; whole wheat pasta; rolled oats; white rice; baked potato	11
Refined, processed grains and starches	Cold breakfast cereal; other cooked breakfast cereal; white bread; bagels, english muffins, or rolls; muffins or biscuits; pasta; tortillas; pancakes or waffles; french fried potatoes; potatoes (baked/boiled/mashed); potato or corn/tortilla chips; crackers; pretzels; breakfast bars; energy bars; low carb bars; pizza	White bread; banana or zucchini bread; white tortilla, muffin, biscuit, or bagel; white pasta; french fried potatoes; mashed potatoes; potato or corn/tortilla chips; granola bar; crackers; pizza; pancake or waffle	12
Unprocessed nuts	Peanuts; walnuts; other nuts	Unsalted peanuts, almonds, walnuts, or pecans	13
Processed nuts	Peanuts; walnuts; other nuts; peanut butter	Salted or candied peanuts, almonds, walnuts or pecans; peanut or almond butter	14
Dairy	Skim milk; 1% or 2% milk; whole milk; soy milk; cream; coffee whitener; flavored yogurt; yogurt; cottage or ricotta cheese; cream cheese; other cheese	Milk, yogurt, cream, cheese	15
Chocolate or candy	Milk chocolate; dark chocolate; candy bars (e.g. Snickers, Milky Way, Reeses); candy (e.g. mints, Lifesavers)	Milk chocolate; dark chocolate; candy bars (e.g. Snickers, Milky Way, or Reeses); candy (e.g. Lifesavers, gummy bears, or fruit snacks)	16
Grain-based desserts	Cookies (fat-free or reduced fat), cookies (other); cookies (home baked); brownies; doughnuts; sweet roll, coffee cake, or pastry (reduced-fat); sweet roll, coffee cake or pastry (ready-made); sweet roll, coffee cake, or pastry (home baked); cake (home baked);	Cookies; brownies; cake; pie; doughnuts; sweet rolls	17

	cake (ready-made); pie (homemade); pie (ready-made);		
Dairy-based desserts	Frozen yogurt, sherbet, low-fat ice cream; regular ice cream	Ice cream; milkshake; flan; mousse	18
Sugar-sweetened beverages	Carbonated beverage with caffeine and sugar; other carbonated beverage with sugar; sugared beverages (e.g. punch, lemonade, sports drink, sugared tea)	Soda, sweet tea, sweetened fruit juice, sports drink (e.g. Gatorade)	19
Sweeteners	Jams, jellies, preserves, syrup, or honey; sugar	Jam; jelly; preserves; syrup; sugar; honey	20
Coffee	Decaffeinated coffee, coffee with caffeine, dairy coffee drink	Coffee	21
Vitamins & minerals	Multi-vitamin; beta carotene; vitamins A, B_6, C, D, E; selenium; iron; zinc; calcium or dolomite; fish oil; potassium	Not included	
Miscellaneous, not included	Pure butter; spreadable butter; margarine or spread; low-calorie beverages (with or without caffeine); beer; light beer; red wine; white wine; liquor; water; herbal tea; caffeinated tea; oat bran, other bran, wheat germ; salt; splenda; artificial sweetener; low-fat mayonnaise; mayonnaise; salad dressing; olive oil	Not included	

*Five food group items contained foods from more than one NOVA category. First minimally processed vegetables were categorized as such if they were raw or if they were minimally cooked (steamed, baked, or sautéed). Next, the question concerning processed meat included ground meat in addition to canned and cured meat; the former was not separated into its own category for UPF meats. Third, the item evaluating processed grain consumption as processed calories included whole grain bread and bagels, which can be categorized either as PF or UPF depending on the method of preparation. While the item considering consumption of sweeteners included both group II and IV foods, calories for this question were in the ultra-processed group. Finally, as dairy foods come in all levels and varieties of processing, they were not categorized into a NOVA category and were not included the main analyses of calories were categorized using the NOVA classification system as defined by Monteiro et. al. The survey was designed to discriminate between foods at different levels of processing. Table A1: Food groups and items included in the original FFQ and adapted FFQ.
