# Supplementary material for: Changes in Ultra-Processed Food Consumption and Lifestyle Behaviors Following COVID-19 Shelter-in-Place: A Retrospective Study
Source: Foods. 2021 Oct 23;10(11):2553. doi: 10.3390/foods10112553 (PMC8619493; doi:10.3390/foods10112553)
Supplement: Supplementary file 1 [file foods-10-02553-s001.zip › Supplemental Table S2.pdf]

Table S2. Serving Conversion Chart from Adapted Food Frequency Questionnaire.

Servings in Question	Midpoint Servings/month	Midpoint Servings/day	Low Servings/day	Upper Servings/day
Never	0.00	0.00	0.00	0.00
1-4 Monthly	2.50	0.08	0.03	0.13
2-6 weekly	17.14	0.57	0.29	0.86
1-2 daily	45	1.50	1.00	2.00
3+ daily	120.00	4.00	3.00	5.00
