# Supplementary material for: Changes in Ultra-Processed Food Consumption and Lifestyle Behaviors Following COVID-19 Shelter-in-Place: A Retrospective Study
Source: Foods. 2021 Oct 23;10(11):2553. doi: 10.3390/foods10112553 (PMC8619493; doi:10.3390/foods10112553)
Supplement: Supplementary file 1 [file foods-10-02553-s001.zip › Supplemental Table S3.pdf]

Table S3. Caloric density per survey or FFQ item.

Question	Level of Processing	Average Calories per serving, Calculated
Fresh or frozen fruit	Minimally Processed	62
Min processed fruit	Minimally Processed	86
Raw vegetables	Minimally Processed	27
Unprocessed Lean Protein	Minimally Processed	96
Unprocessed Red Meat	Minimally Processed	176
Whole, Unprocessed Grains	Minimally Processed	252
Unsalted Nuts	Minimally Processed	181
Coffee	Minimally Processed	3.6
Fruit w/ sugar	Processed	87
Canned Vegetables	Processed	55
Processed meat	Processed	182
Whole, Processed Grains	Processed	149
Salted / sugared Nuts	Processed	162
Meat Alternatives	Ultra-processed	183
Processed, Refined Grains	Ultra-processed	182
Chocolate Candy	Ultra-processed	128
Grain-based desserts	Ultra-processed	185
Dairy-based desserts	Ultra-processed	199
Sugar-sweetened beverages	Ultra-processed	135
Sweeteners	Ultra-processed	55
Dairy	N/A	108
