# Supplementary material for: Changes in Ultra-Processed Food Consumption and Lifestyle Behaviors Following COVID-19 Shelter-in-Place: A Retrospective Study
Source: Foods. 2021 Oct 23;10(11):2553. doi: 10.3390/foods10112553 (PMC8619493; doi:10.3390/foods10112553)
Supplement: Supplementary file 1 [file foods-10-02553-s001.zip › Supplemental Table S4.pdf]

Table S4. Univariate linear regression outputs.

	Change in % MPF`		Change in % PF		Change in % UPF		Weight Change	
**Coefficient**	**estimate**	**P value**	**estimate**	**P value**	**estimate**	**P value**	**estimate**	**P value**
Δ Hours Spent Outside	-0.04	0.670	-0.09	0.078	0.13	0.147	-0.03	0.686
Bachelor's Degree	-0.47	0.241	0.16	0.469	0.31	0.406	0.35	0.315
Graduate Degree	-0.03	0.936	0.03	0.880	0.00	0.999	0.03	0.933
Effect of Male	0.43	0.183	0.05	0.783	-0.47	0.117	-0.54	0.056
Presence of Children	0.60	0.111	-0.15	0.448	-0.45	0.208	0.07	0.828
Mental Health	-0.46	0.119	-0.05	0.750	0.51	0.067	0.54	0.037
Household Income	-0.12	0.281	0.06	0.302	0.06	0.578	-0.05	0.638
Baseline Exercise	0.01	0.960	-0.08	0.361	0.07	0.642	0.06	0.688
Δ Takeout	-0.25	0.016	0.02	0.748	0.24	0.018	0.22	0.018
Δ Pre-Packaged Food	-0.73	0.000	0.16	0.029	0.57	0.000	0.80	0.000
Δ Cooking	0.50	0.000	-0.14	0.050	-0.36	0.004	-0.39	0.001
Δ Snacking	-1.18	0.000	0.13	0.092	1.05	0.000	1.33	0.000
Δ Sedentary Activity	-0.65	0.000	0.20	0.009	0.44	0.001	0.94	0.000
Δ Alcohol Consumption	-0.60	0.000	0.12	0.118	0.47	0.001	0.70	0.000
Absolute Δ MPF							0.14	0.235
Absolute Δ PF							0.78	0.000
Absolute Δ UPF							1.08	0.000

Abbreviations: Minimally-processed food (MPF); processed food (PF); ultra-processed food (UPF); change (Δ).
