# Supplementary material for: Changes in Ultra-Processed Food Consumption and Lifestyle Behaviors Following COVID-19 Shelter-in-Place: A Retrospective Study
Source: Foods. 2021 Oct 23;10(11):2553. doi: 10.3390/foods10112553 (PMC8619493; doi:10.3390/foods10112553)
Supplement: Supplementary file 1 [file foods-10-02553-s001.zip › Supplemental Table S5.pdf]

Table S5. Stepwise linear regression outputs.

	Change in % MPF		Change in % PF		Change in % UPF		Weight Change	
**coefficient**	**estimate**	**P value**	**estimate**	**P value**	**estimate**	**P value**	**estimate**	**P value**
Δ Hours Inside			-0.09	0.078	0.16	0.068		
Effect of Male					-0.51	0.094	-0.51	0.067
Presence of Children	0.56	0.140						
Mental Health	-0.42	0.151			0.54	0.051	0.51	0.045
Δ Takeout							-0.15	0.092
Δ Pre-Packaged Food	-0.26	0.066			0.21	0.106	0.35	0.002
Δ Cooking	0.38	0.004	-0.16	0.027	-0.23	0.064	-0.25	0.022
Δ Snacking	-0.91	0.000			0.96	0.000	0.76	0.000
Δ Sedentary Activity	-0.28	0.052	0.22	0.005			0.50	0.000
Δ Alcohol Consumption	-0.25	0.074					0.30	0.008
Absolute Δ UPF							0.66	0.000

Abbreviations: Minimally-processed food (MPF); processed food (PF); ultra-processed food (UPF); change (Δ).
