# Supplementary material for: Changes in Ultra-Processed Food Consumption and Lifestyle Behaviors Following COVID-19 Shelter-in-Place: A Retrospective Study
Source: Foods. 2021 Oct 23;10(11):2553. doi: 10.3390/foods10112553 (PMC8619493; doi:10.3390/foods10112553)
Supplement: Supplementary file 1 [file foods-10-02553-s001.zip › Supplementary Methods 21.08.26.pdf]

Changes in Ultra-Processed Food Consumption and Lifestyle Behaviors Following COVID-19 Shelter-In-Place: A Retrospective Study

Supplementary Methods

Non-food Survey Questions

1. How tall are you? (feet & inches)
2. How much do you weigh in pounds? (Enter response rounded to the nearest pound)
3. What is your sex? (male, female, other)
4. What is your race or ethnicity? (check all that apply)
    - Black, Native American, Asian, Native Hawaiian/Pacific Islander, Hispanic, Caucasian, other
5. What was your monthly household income before the COVID-19 pandemic in dollars? (select one)
    - $1,000>; $1,000-$2,499; $2,500-$4,999; $5,000-$9,999; $10,000-$24,999; $25,000<
6. What is the highest level of education that you have completed? (select one)
    - Did not graduate high school; graduated high school; some college; associate degree; bachelor’s degree; graduate degree
7. Did you spend a significant amount of time caring for one or more children during shelter-in-place? (Y/N)
8. Have you or one of your family members lost a job or a significant source of income due to the Covid-19 pandemic? (Y/N)
9. Did you or a family member have a serious adverse life event such as a COVID-19 hospitalization during the shelter-in-place order? (Y/N)
10. Did you face any mental health challenges such as anxiety or depression **before** the COVID-19 shelter-in-place order? (Y/N)
11. Did you face any new mental health challenges such as anxiety or depression **during** the COVID-19 shelter-in-place order? (Y/N)
12. How many hours per week did you spend outside the house working or engaging in other activities before the Covid-19 shelter-in-place order that lasted from mid-March through May? (select one)
    - 0-10; 10-20; 20-30; 30-40; 40+
13. How many hours per week did you spend outside the house working or engaging in other activities during the Covid-19 shelter-in-place order that lasted from mid-March through May? (select one)
    - 0-10; 10-20; 20-30; 30-40; 40+
14. Do you smoke or vape tobacco products? (Y/N)
15. Do you smoke or otherwise consume marijuana products? (Y/N)
16. How often did you exercise on average before the Covid-19 shelter-in-place order? (select one)
    - Never; 1-4x monthly; 1-3x weekly; 4-7x weekly; >7x weekly
17. How, if at all, has the Covid-19 shelter-in-place order changed your daily activities in comparison to your life before the pandemic? During the shelter-in-place order… (Likert scale questions with possible responses: significantly less; moderately less; at equal frequency; moderately more; significantly more)
    - I ate takeout...

Changes in Ultra-Processed Food Consumption and Lifestyle Behaviors Following COVID-19 Shelter-In-Place: A Retrospective Study

Supplementary Methods

- I ate ready-to-eat packaged food...
- I cooked...
- I snacked in between meals...
- I think I ate… (More or less healthily)
- I spent more/less money per week on food...
- I exercised more/less…
- I engaged in sedentary behavior…
- I drank alcohol...
- I smoked or vaped tobacco…
- I smoked or consumed marijuana...

18. Did you gain or lose weight during the shelter-in-place order? (select one)
    - Lost >10 lbs.; lost 6-10 lbs.; lost 1-5 lbs.; no change; gained 1-5 lbs.; gained 6-10 lbs.; gained >10 lbs.
